# Antenatal Maternal Emotional Distress and Duration of Pregnancy

**DOI:** 10.1371/journal.pone.0101682

**Published:** 2014-07-07

**Authors:** Mirjam Lukasse, Anne Helbig, Jūratė Šaltytė Benth, Malin Eberhard-Gran

**Affiliations:** 1 Department of Public Health and General Practice, Norwegian University of Science and Technology, Trondheim, Norway; 2 Department of Health, Nutrition and Management, Oslo and Akershus university college of applied sciences, Oslo, Norway; 3 Norwegian Resource Centre for Women's Health, Oslo University Hospital, Oslo, Norway; 4 Department of Obstetrics, Oslo University Hospital, Oslo, Norway; 5 Institute of Clinical Medicine, Campus Ahus, University of Oslo, and Health Services Research Centre, Akershus University Hospital, Lørenskog, Norway; 6 Division of Mental Health, Norwegian Institute of Public Health, and Health Services Research Centre, Akershus University Hospital, Lørenskog, Norway; UCL Institute of Child Health, University College London, United Kingdom

## Abstract

**Objective(s):**

We sought to prospectively study the association between antenatal emotional distress and gestational length at birth as well as preterm birth.

**Study Design:**

We followed up 40,077 primiparous women in the Norwegian Mother and Child Cohort Study. Emotional distress was reported in a short form of the Hopkins Symptom Checklist-25 (SCL-5) at 17 and 30 weeks of gestation. Gestational length at birth, obtained from the Medical Birth Registry of Norway, was used as continuous (gestational length in days) and categorized (early preterm (22–31 weeks) and late preterm (32–36 weeks) versus term birth (≥37 weeks)) outcome, using linear and logistic regression analysis, respectively. Births were divided into spontaneous and provider-initiated.

**Results:**

Of all women, 7.4% reported emotional distress at 17 weeks, 6.0% at 30 weeks and 5.1% had a preterm birth. All measurements of emotional distress at 30 weeks were significantly associated with a reduction of gestational length, in days, for provider-initiated births at term. Emotional distress at 30 weeks showed a reduced duration of pregnancy at birth of 2.40 days for provider-initiated births at term. An increase in emotional distress from 17 to 30 weeks was associated with a reduction of gestational length at birth of 2.13 days for provider-initiated births at term. Sustained high emotional distress was associated with a reduction of gestational length at birth of 2.82 days for provider-initiated births. Emotional distress did not increase the risk of either early or late preterm birth.

**Conclusion:**

Emotional distress at 30 weeks, an increase in emotional distress from 17 to 30 weeks and sustained high levels of emotional distress were associated with a reduction in gestational length in days for provider-initiated term birth. We found no significant association between emotional distress and the risk of preterm birth.

## Introduction

Preterm birth (defined as birth before 37 weeks of gestation, WHO 1977) is a common but serious pregnancy complication [1], [2]. The majority of neonatal intensive care admissions are from preterm babies [3], with preterm birth remaining a leading cause of neonatal mortality and morbidity [2], [4].

Since the nineties a growing awareness that maternal antenatal stress may influence gestational length at birth and preterm birth in particular has led to a large number of studies, as the comprehensive review of Dunkel Schetter & Glynn indicates [5]. Studies have investigated the impact of stressors such as major life events [6], daily hassles [7], and disasters [8]. Other studies have assessed the association between preterm birth and emotional states such as pregnancy-related anxiety [9], [10], general anxiety and depression [11], and various measures of psychosocial stress [12].

The influence of maternal psychological distress and stress hormones on the risk of preterm birth is not well defined, although patterns are beginning to emerge [13]. The main pathways proposed for this association are the neuroendocrine system, the immunological system, and behavioural stress responses [5], [14]. Briefly, the hypothalamic-pituitary-adrenal axis is involved in stress responses as well as in the onset of parturition;, this includes the “stress hormones” cortisol and corticotrophin releasing hormone (CRH) whose physiology is significantly affected by pregnancy [14]. Maternal inflammation and infection is one of the main causes of preterm birth [1], [15], and the effects of chronic stress on the immune system are well known [16]. Indirectly, behavioural responses to stress such as smoking and substance abuse may increase the risk of preterm birth [5].

Research on both gestational length at birth and preterm birth are of interest. Using gestational length at birth as a continuous variable adds statistical power and may reveal associations not detected when using cut-offs, such as early and late preterm birth. A shortening of gestational length at birth by a day, or couple of days can be detected using a continuous variable [17]. Recent research has shown that length of gestation at birth matters, also at term [18]. It is recommended to perform term elective caesarean sections after 39 weeks, as doing so provides better neonatal outcomes than before 39 weeks [18]. A growing awareness of the unfavourable effects of late preterm birth has led to the investigation of the role of non-spontaneous preterm birth and indications for this [19], [20]. In the newly presented classification of preterm birth by WHO, non-spontaneous preterm birth is termed “*provider-initiated* ***”*** and defined as induction of labor or elective caesarean section without premature rupture of membranes [21], [22]. A recent study in the Unites States reported that more than half of all provider-initiated preterm births at 34–36 weeks gestation were carried out in the absence of a strong medical indication [20].The potential impact of maternal stress during pregnancy on gestational length at birth and preterm birth through provider-initiated start of labour has had limited attention. Previous studies have either focused on spontaneous preterm birth or not differentiated between spontaneous or provider-initiated birth when examining gestational length at birth [9], [17], [23]–[25].

Another issue, which has been poorly investigated to date, is the role of the pattern of prenatal stress as a possible predictor of reduced gestational age at birth. Research indicates that levels of anxiety and depression vary during pregnancy, with higher levels at the beginning and lower levels at 30 weeks gestation [26], [27]. The only study investigating this so far, by Glynn et al., suggests that a deviation from this pattern, i.e. an increase in perceived stress and anxiety from the second to third trimester could be a better predictor of preterm birth than stress levels at either time point [28].

The Norwegian Mother and Child Cohort (MoBa) is a nationwide population-based prospective cohort study of pregnant women. It included measurements of emotional distress on two occasions in pregnancy as well as a large number of relevant covariates. The aim of our study was to estimate the association between antenatal emotional distress in the second and third trimester, and of a pattern of either increasing or sustained high levels of emotional distress, with gestational length at birth as well as increased risk of preterm birth. All of these associations were assessed for provider-initiated births versus spontaneous births.

## Materials and Methods

The MoBa study, conducted by the Norwegian Institute of Public Health [29], recruited pregnant women from all over Norway from 1999–2008, and 38.5% of invited women consented to participate. Participating women completed two extensive questionnaires at around 17 and 30 weeks' of gestation, including measures of emotional distress. These data were linked to the Medical Birth Registry of Norway (MBRN), providing information on pregnancy and birth outcome. The MoBa study has been described in detail previously [29]. More information can be found at the website of the Norwegian Institute of Public Health (www.fhi.no). The current study is based on version 6 of the quality-assured data files released for research in 2011. Our study included 45,769 primiparous women with a singleton pregnancy who gave birth between 22 and 44 weeks of gestation. We excluded 1,383 women for whom information on emotional distress at 17 weeks was missing completely and 1,302 women who gave birth to a baby with serious congenital malformation(s). Another 118 cases were excluded due to birth weights 3.0 standard deviations (SD) above or 3.5 SD below gender- and gestational age-adjusted national reference values [30], indicating a high risk of incorrect registration of gestational age. Finally, we excluded 2,889 women with the following pre-existent medical risk factors for preterm birth; previous cervical surgery, insulin-dependent diabetes mellitus, hypertension, congenital heart disease, other vascular or heart disease, inflammatory bowel disease, systemic lupus erythematosus, and rheumatoid arthritis (all self-reported in questionnaire 1, at 17 weeks). Thus, our final study sample consisted of 40,077 women.

### Ethics statement

The Regional Committee for Medical Research Ethics in South-Eastern Norway (Regional Committee for Research Ethics in Health Region II, Ref.SAFH 95/313 RTL) and the Norwegian Data Inspectorate approved the study. Informed written consent was obtained from each participant. The protocol for this study was submitted to the Norwegian Institute of Public health who upon approval supplied the researchers of this study with anonymized data through contract (PDB 698, www.fhi.no/moba).The Regional Committee for Medical Research Ethics in South-Eastern Norway (Regional

### Emotional distress

Emotional distress was measured using a short version of the Hopkins Symptom Checklist-25, the Symptom Checklist-5 (SCL-5), at around 17 weeks (mean 17.4, SD ±2.8) and 30 weeks' gestation (mean 30.6, SD ±2.0). Although such a measurement cannot replace a clinical interview, previous research [31] has shown that cut-off value of 2.0 gives the same prevalence estimate of a depressive disorder as a well-known diagnostic interview, the Composite International Diagnostic Interview [32]. The advantage of using the SCL is that it is designed to measure symptoms of depression and anxiety in population surveys [33], [34]. Furthermore, it has been validated in several populations and documented as an acceptable screening instrument for depression as defined by the ICD-10 [31], [35]. The SCL-5 has been used in several previous studies as a measurement for depression [36] and emotional distress [37], [38]. The SCL-5 is highly correlated (r = 0.92) with the SCL-25 [39] and consists of the following questions: Have you been bothered by any of the following during the last two weeks: (1) feeling fearful, (2) nervousness or shakiness inside, (3) feeling hopeless about the future, (4) feeling blue, (5) worrying too much about things. The response categories ranged from ‘not bothered’ to ‘very bothered’ (range 1–4), with a maximum total score of 20. Emotional distress was defined as a mean score of ≥2 [33]. In the current sample, the SCL-5 had adequate internal consistency with a Cronbach's α of 0.81 at both 17 and 30 weeks. There were 367 (0.8%) and 3,904 (8.8%) women with missing values on SCL-5 in pregnancy week 17 and 30, respectively. Missing values in the dichotomized version of the SCL-5 were imputed as follows: first, the average score on existing items was calculated for each case. Only if the average of the existing items was clearly above or below the cut-off and could not be affected by imputed values, were missing values imputed by zero or one as appropriate. Imputation was not performed on cases where the average score was not uniquely defining the value above or below cut-off. Increase in emotional distress was defined as no emotional distress at 17 weeks but present at 30 weeks. This group and the group of sustained emotional distress (i.e. emotional distress present at both time points) were compared to the reference group defined as sustained low or decreasing distress (i.e. women who had emotional distress at neither 17 nor 30 weeks, and women who had emotional distress at 17 but not 30 weeks).

### Duration of pregnancy

The actual and the estimated date of delivery (EDD, determined by ultrasound at approximately 18 weeks) was obtained from the MBRN database, and used to calculate gestational length at birth in days. For the 798 (1.8%) women for whom no ultrasound based EDD was available, gestational length at birth was calculated using the last menstrual period. Preterm birth was defined as birth before 37+0 weeks (259 days) of gestation, as opposed to term birth at or after 37+0 weeks. Preterm birth is often categorized into <28 weeks, 28–<32 weeks and 32–<37 weeks [2], [40], [41]. Due to few births before 28+0 weeks gestation, we defined all births before 32+0 weeks (224 days) of gestation as early preterm birth and those from 32+0 weeks to 36+6 weeks as late preterm birth.

### Covariates

Maternal age, education, marital status, smoking, maternal pre-pregnancy body mass index (BMI, kg/m^2^), and history of spontaneous abortion (<22 weeks gestation) were obtained from the MoBa questionnaire at 17 weeks gestation. The gender and birth weight of the new-born were obtained from the MBRN database. Major fetal malformations were derived from the variable “serious congenital malformations” from MBRN records. We defined a “late medical risk” variable which included risk factors occurring during pregnancy. These consisted of pregnancy-induced hypertensive disease as registered in the MBRN records, self-reported urinary tract infection(s) and episodes of fever after 21 weeks from the MoBa questionnaire at 30 weeks. Since more than 99% of the MoBa participants were of Caucasian origin, ethnicity was not a relevant confounding factor in our study. The variable “start of birth” was used to differentiate between provider-initiated births, i.e. starting with induction of labor or elective caesarean section without preterm rupture of membranes, and spontaneous births, i.e. starting with contractions or spontaneous rupture of the membranes [21].

### Statistical methods and analytical approach

Characteristics of the sample were presented as frequencies and percentages within different categories of the preterm birth variable (Table 1). The outcome gestational length at birth was assessed in two ways: continuous (length in days) and categorized as early preterm birth, late preterm birth, and term birth. Linear and logistic regression models were fitted for the following exposures: emotional distress at 17 weeks, emotional distress at 30 weeks, increase in emotional distress and sustained high emotional distress. Women who gave birth before 32 weeks' gestation were excluded from all analyses, which included the SCL-5 at 30 weeks, to avoid assessing exposure and outcome simultaneously. This means that for the emotional distress variables, other than “emotional distress at 17 weeks”, the preterm group was limited to women delivering at 32+0–36+6 weeks' gestation. All analyses were further adjusted for maternal age, educational level, smoking, pre-pregnancy BMI, a history of spontaneous abortion, late medical risk, newborn gender, and start of birth. In addition, the interaction between how birth started (spontaneous versus provider-initiated) and emotional distress was included into the models. When estimating the association between emotional distress and gestational length at birth we first performed analyses for the whole sample. Considering the results for emotional distress and gestational length at birth for provider-initiated births, we then through stratified analyses proceeded to investigate whether the association between gestational length and emotional distress occurred selectively preterm or at term (Table 2). The interaction between how birth started was not significant in the models assessing the association between emotional distress and early and late preterm birth, categorized and was therefore excluded (Table 3). An interaction between newborn gender and emotional distress was also assessed, but was not significant in any of the models. All analyses were performed with SPSS v20. P-values <0.05 were considered statistically significant.

**Table 1:** Background characteristics and distress measures according to preterm birth.

		Early preterm	Late preterm	Term
		22–31 weeks	32–36 weeks	≥37 weeks
		n = 272	n = 1,763	n = 38,042
		n (%)	n (%)	n (%)
**Emotional distress at 17 weeks**				
Yes		21 (7.7)	125 (7.1)	2,805 (7.4)
No		251 (92.3)	1,638 (92.9)	35,237 (92.6)
**Emotional distress at 30 weeks** *				
Yes		19 (7.0)	105 (6.0)	2,286 (6.0)
No		94 (34.6)	1,484 (84.2)	32,820 (86.3)
Missing		159 (58.5)	174 (9.9)	2,936 (7.7)
**Change in emotional distress**				
Increase		17 (6.3)	61 (3.5)	1,254 (3.3)
Decrease or sustained low		94 (34.6)	1,484 (84.2)	32,820 (86.3)
Sustained high		2 (0.7)	44 (2.5)	1,032 (2.7)
Missing		159 (58.5)	174 (9.9)	2,936 (7.7)
**Answered questionnaire at 30 weeks** *				
Yes		125 (46.0)	1,610 (91.3)	35,582 (93.5)
No		147 (54.0)	153 (8.7)	2,460 (6.5)
**Age (years)**				
	<26	75 (27.6)	520 (29.5)	11,065 (29.1)
	26–30	106 (39.0)	736 (41.7)	16,956 (44.6)
	31–36	74 (27.2)	429 (24.3)	8,797 (23.1)
	>36	17 (6.2)	78 (4.4)	1,224 (3.2)
**Educational level**				
	Primary school	13 (5.1)	53 (3.2)	984 (2.7)
	Secondary/Grammar school	93 (36.2)	607 (36.2)	11,887 (32.8)
	University/college <4 years	96 (37.4)	633 (37.7)	14,506 (40.0)
	University/college ≥4 years	55 (21.4)	384 (22.9)	8,853 (24.4)
**Married or cohabiting**		256 (94.1)	1,663 (94.9)	35,980 (95.0)
**Smoking**		18 (6.6)	160 (9.1)	3,001 (7.9)
**Pre-pregnancy body mass index (kg/m^2^)**				
	<20	33 (12.5)	238 (13.9)	5,295 (14.4)
	20–24	133 (50.2)	935 (54.7)	21,369 (58.0)
	25–29	57 (21.5)	349 (20.4)	7204 (19.6)
	≥30	42 (15.8)	188 (11.0)	2,976 (8.1)
**Medical risk factors**				
	History of spontaneous abortion	56 (20.6)	239 (13.6)	4,951 (13.0)
	Late medical risk	90 (33.1)	483 (27.4)	3,633 (9.5)
**Birth weight (grams)**				
	<1500	199 (74.0)	42 (2.4)	0
	1500–3000	70 (26.0)	1,449 (82.2)	4,172 (11.0)
	>3000	0	272 (15.4)	33,848 (89.0)
**New-born gender**				
	Boy	139 (51.1)	944 (53.5)	19,418 (51.0)
	Girl	133 (48.9)	819 (46.5)	18,624 (49.0)
**Any covariate missing**		32 (11.8)	216 (12.3)	4,656 (12.2)
**Start of birth**				
Spontaneous		157 (42.3)	1,299 (73.7)	32,712 (86.0)
Provider-initiated		115 (42.3)	464 (26.3)	5,330 (14.0)

*mean gestational age for filling out questionnaire 2 was 30 weeks, SD 2 weeks. However, some women filled out the form before 30 weeks' gestation while some gave birth after week 30 (in week 31). As a result, approximately 40% of those giving birth early preterm filled out questionnaire 2 and the SCL-5 a second time, even though they delivered early preterm.

**Table 2:** Adjusted* associations between emotional distress (ED†) and gestational length at birth (days).

	Non-stratified analyses	Stratified for preterm and term birth	
		Preterm<37 weeks n = 1,787‡	Term ≥37 weeks n = 33,386‡
	Coefficient	Coefficient	Coefficient
	(95% CI)	(95% CI)	(95% CI)
**ED at 17 weeks** and spontaneous start of birth	n = 29,993	n = 1,275	n = 28,718
	−0.15 (−0.72; 0.42) P = 0.615	1.00 (−2.74; 4.73) P = 0.600	−0.34 (−0.73; 0.06) P = 0.097
**ED at 17 weeks** and provider-initiated start of birth	n = 5,180	n = 512	n = 4,668
	−0.15 (−0.72; 0.42) P = 0.615	1.00 (−2.74; 4.73) P = 0.600	−0.34 (−0.73; 0.06) P = 0.097
**ED at 30 weeks** and spontaneous start of birth	n = 27,748	n = 1,042	n = 26,706
	−0.07 (−1.15; 1.01) P = 0.899	−1.78 (−3.68; 0.13) P = 0.068	0.003 (−1.07; 1.08) P = 0.996
**ED at 30 weeks** and provider-initiated start of birth	n = 4,668	n = 366	n = 4,302
	−2.32 (−3.54; −1.09) P<0.001	−1.78 (−3.68; 0.13) P = 0.068	−2.40 (−3.40; −1.39) P<0.001
**Increase in ED** and spontaneous start of birth§	n = 27,104	n = 1,016	n = 26,088
	0.37 (−0.61; 1.36) P = 0.456	−2.34 (−4.73; 0.05) P = 0.055	0.51 (−0.55; 1.58) P = 0.346
**Increase in ED** and provider-initiated star of birth§	n = 4,531	n = 358	n = 4,173
	−2.54 (−4.09; −0.99) P = 0.001	−2.34 (−4.73; 0.05) P = 0.055	−2.13 (−3.41; −0.85) P = 0.001
**Sustained high ED** and spontaneous start of birth§	n = 26,862	n = 1,006	n = 25,856
	−0.93 (−1.73; −0.13) P = 0.022	−0.83 (−3.84; 2.18) P = 0.589	−0.72 (−1.69; 0.25) P = 0.145
**Sustained high ED** and provider-initiated start of birth§	n = 4,459	n = 348	n = 4,111
	−0.93 (−1.73; −0.13) P = 0.022	−0.83 (−3.84; 2.18) P = 0.589	−2.82 (−4.37; −1.27) P<0.001

*adjusted for maternal age, educational level, smoking, pre-pregnancy body mass index, history of spontaneous abortion, late medical risk and neonate gender. For all analyses including ED at 30 weeks, the preterm group is limited to women delivering at 32+0–36+6 week's gestation.

† emotional distress at 17 and 30 weeks are dichotomous variables, mean score ≥2 = 1.

‡ cases with covariates missing are excluded.

§ compared to no emotional distress at 30 weeks.

**Table 3:** Crude and adjusted OR for emotional distress and early and late preterm birth.

	Early preterm	Late preterm		Term
	22–31weeks	32–36 weeks		≥37 weeks
	n = 240*	n = 1,547*		n = 33,386*
	*Crude*	*Crude*	*Adjusted* †	*Control*
	*OR (95% CI)*	*OR (95% CI)*	*OR (95% CI)*	
**Emotional distress** ‡ **at 17 weeks**	n = 240	n = 1,547	n = 1,547	n = 33,386
	0.79 (0.44; 1.41) P = 0.425	1.03 (0.83; 1.26) P = 0.818	0.97 (0.78;1.19) P = 0.747	1
**Emotional distress** ‡ **at 30 weeks**		n = 1,408	n = 1,408	n = 31,008
	Not calculated||	1.09 (0.87; 1.36) P = 0.447	1.00 (0.80;1.25) P = 0.993	1
**Increase in emotional distress** §		n = 1,374	n = 1,374	n = 30,261
	Not calculated||	1.15 (0.87; 1.52) P = 0.331	1.06 (0.80; 1.41) P = 0.671	1
**Sustained high ED** §		n = 1,354	n = 1,354	n = 29,967
	Not calculated||	1.01 (0.71; 1.43) P = 0.966	0.93 (0.66; 1.33) P = 0.696	1

*cases with covariates missing are excluded.

† adjusted for maternal age, educational level, marital status, smoking, pre-pregnancy body mass index, late medical risk, history of spontaneous abortion, and neonate gender.

‡ emotional distress at 17 and 30 weeks are dichotomized variables, mean score ≥2 = 1.

§ compared to no emotional distress at 30 weeks.

|| OR not calculated because the exposure was measured simultaneously with the outcome variable.

## Results

Table 1 presents background characteristics of the participants by early preterm, late preterm and term birth. Emotional distress was reported by 2,951 (7.4%) women at 17 weeks, and by 2,410 (6.0%) at 30 weeks. Thirteen hundred and thirty two (3.3%) women had emotional distress for the first time at 30 weeks, and 1,078 (2.7%) women had sustained high emotional distress. Emotional distress at 30 weeks was not available for 159 women as they delivered before filling out the second questionnaire. The 113 women, who delivered early preterm, for whom emotional distress data was available, either gave birth between 30 and 32 weeks, or filled out the form before 30 weeks' gestation. Preterm birth occurred in 2,035 (5.1%) women. The median gestational length at birth for the whole study population was 282 days.

Table 2 presents the results for the adjusted associations between emotional distress and gestational length at birth, as a continuous outcome in days (unadjusted results in Table S1). Results are shown for the whole sample, and stratified by preterm and term birth. Emotional distress at 17 weeks was not associated with gestational length in days. Non-stratified analyses showed that emotional distress at 30 weeks was associated with a shortened gestational length of 2.32 days in provider-initiated births. Stratified analyses showed no reduction of gestational length in preterm births, but in term births the association between emotional distress at 30 weeks and gestational length at birth was significant for provider-initiated births. Non-stratified analysis of women who progressed from no emotional distress at 17 weeks to distress at 30 weeks showed a reduction of gestational length at birth of 2.54 days for provider-initiated births. Stratified analyses showed that in women giving birth preterm, this association was not significant. At term, this association was only significant for provider-initiated births (-2.13 days). Sustained high emotional distress at 17 and 30 weeks was associated with a reduction of gestational length at birth of 0.93 days. However, after stratification, this association was present only in provider-initiated births at term (-2.82 days).

Table 3 presents the results for the association between antenatal maternal emotional distress and preterm birth, categorized into early preterm, late preterm and term birth. Neither emotional distress at 17 nor at 30 weeks was not associated with early or late preterm birth. In all analyses, neither increasing nor sustained high emotional distress were associated with an increased risk of preterm birth (Table 3). There was no significant interaction between how birth started and emotional distress for the analyses in table 3.

## Discussion

All our measurements of emotional distress at 30 weeks, but not at 17 weeks, were associated with a reduction in the duration of pregnancy at term, but only in provider-initiated births. The largest reduction of gestational length for term births was seen in women with sustained high emotional distress. We found no significant association between emotional distress and preterm birth for either continuous or categorical outcome measures.

Our study has several strengths. We used an instrument designed to measure symptoms of anxiety and depression in population surveys [33]. It has been validated in several populations and is documented as an acceptable screening instrument for depression as defined by the ICD-10 [31]. Measuring emotional distress twice during pregnancy made it possible to investigate associations for both an increasing and sustained high levels as well as assessing the separate time points. Measuring emotional distress as early as 17 weeks provided information on the possible association with early preterm birth. It strengthens our study that information on many relevant covariates was collected prospectively. The large sample size allowed for control of many known risk factors associated with preterm birth [42], [43]. Finally, the Norwegian setting is unique; in contrast to many other countries, the prevalence of preterm birth in the Norwegian population has remained low and stable, around 6% during the time the MoBa data were collected [40]. The homogeneity of the population may make it easier to isolate the role of emotional distress.

However, our study also has limitations. The homogeneity of our population, for example for known risk factors such as race/ethnicity may limit the generalizability of our findings to other populations. The low participation rate in the Norwegian Mother and Child Cohort study is a problem [29]. The cohort has an under-representation of the youngest women (<25 years), smokers, and women with stillbirths and neonatal deaths [44]. The prevalence of preterm birth in this cohort is also slightly lower than in the general population, most likely due to the socioeconomic gradient [29], [44]. Hence, the women included in our sample may not be representative for the general population. However, a recent study on potential biases of a skewed selection in the Norwegian Mother and Child Cohort found the prevalence estimates, but not the exposure-outcome associations, to be influenced by selection [44].

We found no association between emotional stress measured with the SCL-5 and preterm birth. This lack of an association in our study does not exclude the association between extreme stress after traumatic events and preterm birth as reported by others [6], [45]. Not finding an association between emotional distress and preterm birth could in part be due to our measuring instrument; while acceptable as a screening instrument for depression, its validity for detecting anxiety is rather low [31], [35]. In their comprehensive review Dunkel Schetter & Glynn conclude that anxiety rather than depressed mood is associated with preterm birth [5].

Unexpectedly, we found a significant interaction between emotional distress and how birth started. No previous study has investigated this association; they either do not provide information on how birth started [17], [46], [47] or exclude provider-initiated births [9], [23]. Further investigation of our data showed that this interaction only was significant for term births. We can only speculate what mechanism lies behind the association between emotional distress and gestational length in provider-initiated term births. Hypothetically, this finding could be due to an increased risk of developing obstetric complications in women with emotional distress. Alternatively, despite our controlling for a number of known risk factors and complications, other conditions leading to emotional distress as well as to health providers intervening at term may have been present. Perhaps most likely, women with higher distress levels might more often request an elective caesarean section or induction of labor. We did not have the necessary information to investigate this possibility.

We compared increasing and sustained emotional distress to decreasing or no stress at either 17 or 30 weeks, as the former patterns deviate from the usual trajectory of distress in pregnancy. Earlier studies have reported a decline in maternal distress towards 30 weeks gestation [26], [27], [48], [49]. Glynn et al. found an association between an increase in stress and anxiety during pregnancy and risk of preterm birth in a study of 415 women including 38 preterm births [28]. Our findings do not confirm increasing distress as a risk factor for late preterm birth. This discrepancy could be due to the fact the Glynn et al. used instruments specifically assessing stress and anxiety, while the SCL-5 primarily measures depressive symptoms [31], [35].

We measured emotional distress using a questionnaire, not an interview, and cannot account for the cause of emotional distress in our cohort. Many factors such as life events, personality traits, vulnerabilities or psychosocial factors could play a role, either in isolation or in a more complex interplay with each other. It is important for clinicians to be aware that women with higher emotional distress levels in the third trimester may have shorter pregnancies, especially since health providers seem to play a role in this association. Our findings only affected term births, and reducing gestational age at term by three days hardly constitutes a major health risk for the neonate. However, if maternal emotional distress leads to an increase in elective inductions and caesarean sections – either because of stress-induced complications, or for psychological reasons – this may constitute a health risk for the mother, the neonate, and possibly affect future pregnancies. For this, as well as for general physical and mental health concerns, stress reduction in pregnancy may be of benefit.

## Supporting Information

Table S1 Unadjusted associations between emotional distress and gestational length at birth (days).
(DOCX)
