# Supplementary material for: Antenatal Maternal Emotional Distress and Duration of Pregnancy
Source: PLoS One. 2014 Jul 7;9(7):e101682. doi: 10.1371/journal.pone.0101682 (PMC4084954; doi:10.1371/journal.pone.0101682)
Supplement: Table S1 — Unadjusted associations between emotional distress and gestational length at birth (days). (DOCX) [file pone.0101682.s001.docx]

**Table S1.** Unadjusted associations between emotional distress (ED†) and gestational length at birth (days)

|  | **Non-stratified analyses** | **Stratified for preterm and term birth** | |
| --- | --- | --- | --- |
|  |  | **Preterm** | **Term** |
|  |  | **n<37 weeks* =1,787^‡^** | **≥37 weeks n=33,386^‡^** |
|  | **Coefficient** | **Coefficient** | **Coefficient** |
|  | **(95% CI)** | **(95% CI)** | **(95% CI)** |
| **ED at 17 weeks** | -0.41 (-0.98; 0.16) P=0.160 | 0.43 (-3.30; 4.15) P=0.822 | -0.47 (-0.86; -0.07) P=0.021 |
| **ED at 30 weeks** | -0.68 (-1.20; -0.15) P=0.011 | -1.64 (-3.54; 0.26) P=0.090^\|^ | -0.50 (-0.92; -0.08) P=0.021 |
| **Increase in ED from 17 to 30 weeks**^§^ | -0.33 (-1.00; 0.35) P=0.345 | -2.31 (-4.71; 0.08) P=0.058 | -0.01 (-0.56; 0.54) P=0.980 |
| **Sustained high ED**^§^ | -1.17 (-1.97; -0.37) P=0.004 | -0.57 (-3.55; 2.42) P=0.710 | -1.19 (-1.83; -0.54) P<0.001 |

*For all analyses including ED at 30 weeks, the preterm group is limited to women delivering at 32+0–36+6 weeks.

† emotional distress at 17 and 30 weeks are dichotomous variables, mean score ≥2 = 1

‡ cases with covariates missing are excluded

§ compared to no emotional distress at 30 weeks
